# Delayed surgery for acute type A aortic dissection: a retrospective review of an alternative surgical strategy in the COVID-19 era

**DOI:** 10.1186/s13019-024-02682-2

**Published:** 2024-04-20

**Authors:** Rin Itokawa, Ryosuke Kowatari, Yuki Imamura, Hanae Sasaki, Norihiro Kondo, Kazuyuki Daitoku, Masahito Minakawa

**Affiliations:** Department of Thoracic and Cardiovascular Surgery, Hirosaki University School of Medicine, 5 Zaifu-Cho, Hirosaki, Aomori 036-8562 Japan

**Keywords:** Acute type A aortic dissection, COVID-19 pandemic, Delayed surgery

## Abstract

**Background:**

During the coronavirus disease (COVID-19) pandemic, medical resources have often been limited to emergency surgeries. This study aimed to evaluate our experience with delayed surgery for acute type A aortic dissections (ATAADs).

**Methods:**

A retrospective study was conducted on 33 patients who underwent surgery for ATAADs between January 2020 and December 2021. The patients were divided into two groups: patients treated within 12 h of arrival (E group; *N* = 21) and those treated > 12 h after arrival (D group; *N* = 12) with strict antihypertensive therapy until surgery.

**Results:**

The plasma fibrinogen levels on arrival were lower in the D group than in the E group (174.3 ± 109.1 vs 293.4 ± 165.4, *p* = 0.038). The time to surgery from symptom onset was longer in the D group than in the E group (4 ± 1 h vs. 86 ± 108 h, *p* < 0.001). There was one case (3%) of mortality and seven cases (21%) of cerebral infarctions in the E group. There was no significant difference in the intraoperative data and quantity of blood transfused between the two groups.

**Conclusion:**

Thus, delayed surgery for ATAAD with appropriate preoperative management may be an alternative surgical strategy in the COVID-19 era.

## Background

**Fig. 1:**
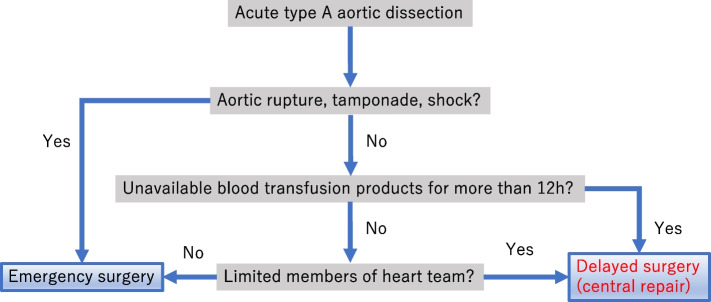
The treatment algorithm for acute aortic dissection during the study period at our institution

Acute type A aortic dissection (ATAAD) is a lethal condition. Despite advances in surgical techniques and perioperative management, operative mortality rates remain at 10.5% in Japan [1]. Emergency surgery is recommended to save patients with ATAAD. However, in the coronavirus disease (COVID-19) era, medical resources, including intensive care units (ICUs), blood transfusions, and the availability of cardiovascular specialists, were limited. There was a serious shortage of blood transfusions, especially due to a decrease in blood donors during the COVID-19 pandemic [2, 3]. Our hospital also had difficulty obtaining platelet products. Therefore, we adopted the strategy of delaying surgery in patients considered stable and capable of waiting until sufficient transfusion products were available. However, we were unclear whether this alternative strategy affected patient prognoses. This retrospective study aimed to evaluate the short-term outcomes of patients with ATAAD who underwent surgery more than 12 h after onset, considered delayed according to this study, and to clarify the surgical outcomes by comparing them with those in whom surgery was not delayed Fig. 1.

## Materials and methods

### Ethical statement

This study was approved by the Hirosaki University Graduate School of Medicine Institutional Review Board (2021–284); the requirement for individual informed patient consent was waived owing to the retrospective nature of the study.

### Patients

Between January 2020 and December 2021, 33 surgeries for ATAAD were performed at our institute. Twenty-one patients were treated within 12 h of onset (E group), and 12 patients were treated > 12 h after onset (D group).

Our principal strategy is to perform emergency surgery for treating ATAAD and save and extend lives; however, surgery was delayed for specific patients, according to our algorithm (Fig. 1). The two chief surgeons (Masahito Minakawa and Norihiro Kondo) decided to delay the surgery depending on hemodynamic stability (without aortic rupture or tamponade), and blood availability (> 12 h). Patients with ATAAD immediately received calcium channel blockers (via continuous intravenous infusion) and β-blockers (via intravenous injection) as antihypertensive therapy. Blood pressures were strictly controlled with a target systolic blood pressure of 90–120 mmHg and a heart rate below 60 bpm in the ICU.

### Surgical technique

Median sternotomies were performed under general anesthesia. The arterial cannulation site was determined based on the patient’s condition, including the presence of cerebral or limb ischemia. Perfusion from the femoral or axillary arteries was performed. Bicaval cannulation and left ventricular venting through the right superior pulmonary vein were performed in all patients. Systemic circulatory arrest was achieved in a state of moderate hypothermia (urinary bladder temperature, 25 °C). Antegrade-selective cerebral perfusion was performed in all patients including ascending aortic replacement cases. The distal anastomosis site was determined based on the patient’s condition. After completion of distal anastomosis with or without frozen elephant trunk, lower-body perfusion was reinstituted through a branch of the graft. Then, proximal anastomosis was performed, after which the arch vessels were reconstructed in the following order: left subclavian artery, left carotid artery, and brachiocephalic artery.

### Statistical analyses

Data were processed using IBM SPSS Statistics software, version 26 (IBM, Armonk, NY, USA). All continuous values are expressed as means ± standard deviations or medians, minimum and maximum. Categorical variables are expressed as patient number (%). Pearson’s chi-squared test or the Mann–Whitney U test was used to compare categorical variables. Differences were considered statistically significant at *p* < 0.05.

## Results

Table 1 presents the characteristics of all the patients analyzed in this study. All patients in this study had negative PCR tests for COVID-19. Preoperative characteristics and comorbidities were comparable between the two groups, except for the plasma fibrinogen levels on arrival, which were lower in the D group than in the E group (174.3 ± 109.1 vs 293.4 ± 165.4, *p* = 0.038). D group did not include tamponade cases. D group included two moderate aortic regurgitation cases, but no severe aortic regurgitation cases. Table 2 lists the operative variables. In E group we had 17 patients with DeBakey Type I dissection meaning arch involvement and sum of patients underwent (partial, Hemi-, or total arch replacement) (1 + 5 + 7) equals 13. Also, in the delayed group we had 11 patients with DeBakey Type I and sum of patients (0 + 1 + 5) equals 6. All 4 patients with DeBakey Type II underwent ascending aortic replacement. In the D group, one coronary ischemic case waited surgery for approximately 21 h because there was no blood transfusion. Catheter intervention was considered, but the patient did not reach myocardial infarction until the surgery. There were two visceral ischemia cases. One visceral ischemia case underwent an exploratory laparotomy and waiting for surgery for DAA since it was confirmed that there was no intestinal necrosis. Fourteen hours after the onset of the disease, a blood transfusion was ready, and he underwent surgery. The other visceral ischemia case underwent urgent thoracic endovascular aneurysm repair. For unknown reason, he had a fever on admission and a positive blood culture, so surgery was performed 5 days later. The operative times, cardiopulmonary bypass times, selective cerebral perfusion times, and cardiac arrest times were comparable between the two groups. The main surgeries and concomitant procedures were also not significantly different between the two groups. The time to surgery from symptom onset was significantly longer in the D group than in the E group (4 ± 1 h vs. 86 ± 108 h, *p* < 0.001). Resection of the proximal entry site was achieved in 90% (19 of 21 patients) of patients in the E group and 91% (11 of 12 patients) of patients in the D group. There was no significant difference in the quantity of blood transfused between the two groups.

**Table 1:** Baseline characteristics

	E group (*n* = 21)	D group (*n* = 12)	*p*-value
Age, years	64.7 ± 14.6	61.1 ± 7.2	0.261
Male, n	12 (57.1%)	8 (66.7%)	0.590
Chronic kidney disease	5 (23.8)	4 (33.3)	0.555
Marfan syndrome	1 (4)	0 (0)	0.443
DeBakey's classification, n (%)			
I	17 (81.0)	11 (91.7)	0.865
II	4 (19.0)	1 (8.3)	0.409
Complete thrombosed FL	1 (4.7)	2 (16.7)	0.252
Preoperative comorbidity, n (%)			
Cardiac tamponade	5 (23.8)	0 (0)	0.067
Moderate aortic regurgitation	2 (9.5)	2 (16)	0.545
Coronary ischemia	2 (9.5)	1 (8.3)	0.909
Cerebral ischemia	2 (9.5)	2 (16.7)	0.545
Renal ischemia	2 (9.5)	3 (25.0)	0.233
Visceral ischemia	1 (4.7)	2 (16.7)	0.252
Limb ischemia	2 (9.5)	1 (8.3)	0.909
Platelets on arrival, × 10^3/μL	18.3 ± 8.3	17.4 ± 6.3	0.613
Preoperative platelets, × 10^3/μL	18.3 ± 8.4	18.1 ± 9.5	0.765
Fibrinogen on arrival, mg/dL	293.4 ± 165.3	174.3 ± 109.1	0.038
Preoperative fibrinogen, mg/dL	293.4 ± 165.4	408.8 ± 299.2	0.379

**Table 2:** Operative variables

	E group (*n* = 21)	D group (*n* = 12)	*p*-value
Onset to operation, h	4 ± 1	86 ± 108	< 0.001
Cardiopulmonary bypass time, min	224 ± 72	257 ± 102	0.500
SCP time, min	98.8 ± 55.8	93.7 ± 71.9	0.653
Cardiac arrest time, min	117 ± 53	136 ± 87	0.852
Minimum bladder temperature (℃)	24.1 ± 2.1	24.4 ± 1.4	0.722
Operative procedure			
Ascending aorta replacement	8 (38.0)	6 (50.0)	0.506
Hemiarch replacement	1 (4.7)	0 (0)	0.443
Partial arch replacement	5 (23.8)	1 (8.3)	0.268
Total arch replacement	7 (33.3)	5 (41.7)	0.632
Concomitant surgery, n (%)			
Aortic root replacement	2 (9.5)	3 (25.0)	0.233
CABG	2 (9.5)	2 (16.7)	0.545
Carotid artery reconstruction	1 (4.8)	2 (16.7)	0.252
F-F crossover bypass	1 (4.8)	1 (8.3)	0.679
Frozen elephant trunk	7 (33.3)	4 (33.3)	1
Site of primary entry site, n (%)			
Ascending aorta	11 (52.4)	8 (66.7)	0.424
Aortic arch	12 (57/1)	4 (33.3)	0.188
Descending aorta	0 (0)	2 (16.7)	0.054
Primary entry resection	19 (90.4)	11 (91.7)	0.909
Transfusion amount			
Red blood cells, unit	3.9 ± 3.1	5.7 ± 8.5	0.789
Fresh frozen plasma, unit	8.3 ± 5.1	11.2 ± 12.1	0.769
Platelet, unit	16.2 ± 5.9	17.5 ± 11.4	0.651
Fibrinogen, mL	14.3 ± 45.1	41.7 ± 66.9	0.112

Values are presented as means ± standard deviations or numbers (%)

*CABG* Coronary artery bypass grafting, *CPB* Cardiopulmonary bypass

The early outcomes are presented in Table 3. The overall mortality rate was 3% (one case out of 33); the deceased patient belonged to the E group and died within 30 days due to sepsis. The overall cerebral infarction rate was 21% (seven cases out of 33; all belonged to the E group). No cerebral infarctions were observed in the D group. There was no difference in the plasma platelet count immediately after the procedure between the two groups.

**Table 3:** Postoperative data

	E group (*n* = 21)	D group (*n* = 12)	*p*-value
In-hospital mortality	1 (4.8)	0 (0)	0.443
Intubation time, h median (minimum–maximum)	36 (14–108)	28 (16–67)	0.866
ICU stay, days	6.8 ± 4.8	7.2 ± 5.1	0.618
Hospital stay, days	21.2 ± 9.7	25.1 ± 12.0	0.431
Morbidities			
Cerebral infarction	7 (33.3)	0 (0)	0.024
Renal failure	2 (9.5)	1 (8.3)	0.909
Re-exploration for bleeding	0 (0)	1 (8.3)	0.179
Postoperative platelets (immediately after the procedure), × 10^3/μL	11.5 ± 3.1	11.1 ± 4.2	0.369
Postoperative fibrinogen (immediately after the procedure), mg/dL	269.6 ± 82.2	312.2 ± 92.3	0.139

Values are presented as means ± standard deviations, numbers (%), or medians (interquartile ranges)

## Discussion

In this study, we analyzed our experience with delayed surgery for ATAAD during the COVID-19 era. Twelve patients underwent surgery > 12 h after ATAAD onset. There was no difference in the early mortality or blood transfusion amounts between the two groups. Our results suggest that strict clinical management in the ICU can be an alternative strategy for ATAAD in the COVID-19 era, allowing for delayed surgery for a limited time.

Surgical treatment of ATAAD remains an effort-intensive procedure despite advances in surgical techniques, cardiopulmonary bypass, clinical diagnosis using computed tomography, and perioperative management. To date, several high-volume studies have reported a related hospital mortality rate of approximately 10% [4, 5]. The mortality rate after ATAAD onset reportedly increases by 1%–2% per hour. The mortality rate with medical treatment is 20% within 24 h, 30% within 48 h, 40% in 7 days, and 50% in 1 month after the onset of symptoms, highlighting the necessity for prompt surgical treatment [6]. Meanwhile, several reports of delayed surgery for ATAAD have been published. For instance, Fukuda et al. [7] reported that intentional delay of surgery in patients with ATAAD and cerebral infarction might be useful. Reportedly, surgical repair should be delayed in patients with ATAAD with malperfusion until the reperfusion injury has resolved [8]. Hamad et al. [9] also reported ATAAD cases with direct oral anticoagulant administration, in which patients were treated medically until coagulation normalized and were successfully treated with surgery. In the COVID-19 era, limitations experienced by cardiovascular teams and restricted availability of blood have compromised timely ATAAD treatment [2]. Therefore, surgeons may have to consider temporary delays until adequate resource planning is achieved. With our strategy, all cases without severe aortic regurgitation or cardiac tamponade were safely treated with delayed surgery.

Delaying surgery for ATAAD may result in increased mortality and blood transfusion due to coagulopathy. ATAAD can result in an increase coagulation capacity and secondarily activate the fibrinolytic system, consuming coagulation factors, fibrinogen, and platelets [10, 11]. In our study, although the plasma fibrinogen levels on arrival were lower in the D group than in the E group, the plasma fibrinogen levels and platelet levels on the last preoperative day were comparable between the two groups. In addition, there were no differences in using fresh frozen plasma or platelet products. As for mortality, the overall 30-day mortality rate in this study was 3%, comparable to recent single-center reports of in-hospital deaths ranging from 2.8% to 4.7% [12–14]. Regarding complications, E group had more tamponade cases and other urgent cases, which may have led to a higher incidence of cerebral infarction than D group.

Despite decreases in blood supply, transfusion continues for emergency surgeries, which have a high demand for blood [15]. While the cessation of elective procedures has reduced the demand for blood products, platelet demand has remained high [16]. Importantly, our strategy was adopted because of the limitations of medical resources during the COVID-19 pandemic, and we found favorable results. Transfusions amounts tended to be higher in D group but without statistically difference. Our pilot study highlights the feasibility and usefulness of delayed ATAAD surgery in certain patients when conditions are not favorable, such as during pandemics. More research is necessary to ascertain if ATAAD patients with specific clinical characteristics can be treated with delayed surgery.

Our study has the inherent limitations of being a single-institution retrospective study conducted on a small number of non-homogeneous patients. Furthermore, patients' conditions and medical treatment may have varied based on the severity of the pandemic during treatment. Further multicenter clinical studies are warranted to explore the utility and efficacy of delayed surgery for ATAAD treatment.

## Conclusions

Delayed surgery in patients with ATAAD who can undergo appropriate preoperative management in the ICU may not increase mortality. Delayed surgery could be an alternative surgical strategy for ATAAD during the COVID-19 era.

## Data Availability

The data that support the findings of this study are available upon request. Due to privacy and confidentiality restrictions, the raw data cannot be publicly shared. However, summarized and anonymized data can be made available to researchers who meet the criteria for access.

## Abbreviations

ATAAD: Acute type A aortic dissection
COVID-19: Coronavirus disease
ICU: Intensive care unit
